# Curcumin Modulates the Inflammatory Response and Inhibits Subsequent Fibrosis in a Mouse Model of Viral-induced Acute Respiratory Distress Syndrome

**DOI:** 10.1371/journal.pone.0057285

**Published:** 2013-02-20

**Authors:** Sreedevi Avasarala, Fangfang Zhang, Guangliang Liu, Ruixue Wang, Steven D. London, Lucille London

**Affiliations:** Department of Oral Biology and Pathology, School of Dental Medicine, Stony Brook University, Stony Brook, New York, United States of America; University of Pittsburgh, United States of America

## Abstract

Acute Respiratory Distress Syndrome (ARDS) is a clinical syndrome characterized by diffuse alveolar damage usually secondary to an intense host inflammatory response of the lung to a pulmonary or extrapulmonary infectious or non-infectious insult often leading to the development of intra-alveolar and interstitial fibrosis. Curcumin, the principal curcumoid of the popular Indian spice turmeric, has been demonstrated as an anti-oxidant and anti-inflammatory agent in a broad spectrum of diseases. Using our well-established model of reovirus 1/L-induced acute viral pneumonia, which displays many of the characteristics of the human ALI/ARDS, we evaluated the anti-inflammatory and anti-fibrotic effects of curcumin. Female CBA/J mice were treated with curcumin (50 mg/kg) 5 days prior to intranasal inoculation with 10^7^ pfu reovirus 1/L and daily, thereafter. Mice were evaluated for key features associated with ALI/ARDS. Administration of curcumin significantly modulated inflammation and fibrosis, as revealed by histological and biochemical analysis. The expression of IL-6, IL-10, IFNγ, and MCP-1, key chemokines/cytokines implicated in the development of ALI/ARDS, from both the inflammatory infiltrate and whole lung tissue were modulated by curcumin potentially through a reduction in the phosphorylated form of NFκB p65. While the expression of TGFß1 was not modulated by curcumin, TGFß Receptor II, which is required for TGFß signaling, was significantly reduced. In addition, curcumin also significantly inhibited the expression of α-smooth muscle actin and Tenascin-C, key markers of myofibroblast activation. This data strongly supports a role for curcumin in modulating the pathogenesis of viral-induced ALI/ARDS in a pre-clinical model potentially manifested through the alteration of inflammation and myofibroblast differentiation.

## Introduction

Acute Respiratory Distress Syndrome (ARDS), the most severe form of acute lung injury (ALI), is a clinical manifestation of the response of the lung to pulmonary insults brought on by infectious, non-infectious and other damaging events and affects up to 200,000 patients annually in the US with a mortality rate approaching 50% with inflammation and tissue fibrosis being the leading cause of morbidity and mortality [1], [2]. Pulmonary fibrosis, itself, is a devastating disease with an almost universally terminal outcome affecting five million people worldwide, including some 200,000 cases culminating in 40,000 deaths/year in the US [3]. Factors that predispose to ARDS are diverse and include sepsis, aspiration, and pneumonias [1], [2]. Although supportive therapy has improved survival somewhat, there are no effective therapeutic agents for improving clinical outcomes in ARDS patients [1], [2]. Therefore, there is an urgent need for the development of treatments to halt the progression of this devastating syndrome.

There are limited models to study ALI/ARDS and no good model systems currently available to study ALI/ARDS and/or pulmonary fibrosis initiated by an infectious (viral) insult [4]. Pulmonary infection of CBA/J mice with 10^7^ pfu reovirus serotype 1, strain Lang (reovirus 1/L) induces ALI/ARDS, providing a model that recapitulates both its acute exudative phase, including the formation of hyaline membranes, as well as its regenerative phase with healing by repair, leading to intra-alveolar and interstitial fibrosis [5], [6]. As with ARDS in human patients, corticosteroids were ineffective in attenuating the infiltration of inflammatory leukocytes, suppressing key cytokine/chemokine expression, and inhibiting the development of fibrotic lesions in reovirus 1/L-ALI/ARDS [7], [8]. Finally, aberrant apoptosis has been proposed as one mechanism leading to fibrotic lesion development in ALI/ARDS, and we have demonstrated an indirect role for the Fas/FasL pathway in reovirus 1/L-ALI/ARDS [9]. Therefore, our model provides a very clinically relevant model for infection-induced acute viral pneumonia leading to ALI/ARDS.

Curcumin, a natural phytochemical present in turmeric, the ground powder of the rhizomes of *Curcuma longa*, has been described as having anti-oxidant, anti-inflammatory, and anti-carcinogenic properties and has been used to treat cancer, arthritis, digestive and liver abnormalities, and respiratory infections [10]–[16]. Toxicity studies conducted on animals have indicated no adverse effects with prolonged use [14], [17]–[19]. While curcumin's effects have been studied in many diseases, the full potential benefits of curcumin and its mechanisms of action have just begun to be elucidated for the treatment of pulmonary diseases including fibrosis and ALI/ARDS [20]–[28]. Pretreatment with curcumin showed beneficial effects on ALI induced by oleic acid [29], by paraquat toxicity [26], by sepsis-induced ALI [30]–[34] and by aspiration-induced ALI [35]. Although the precise mechanism by which curcumin mediated it's beneficial effects in these model systems is not clear, it has been suggested that the modulation of both pro-inflammatory and anti-inflammatory factors may be involved in curcumin's effects [14], [17]–[19]. In this study, curcumin treatment *in vivo* effected both inflammatory (diffuse alveolar damage, DAD) and fibrotic lesion development leading to a significant reduction in the development of ALI/ARDS in reovirus 1/L-infected mice in this pre-clinical model, which may be manifested, at least in part, through the modulation of cytokine/chemokine expression through NFκB and through modulation of myofibroblast differentiation and fibrosis through the regulation of TGFß receptor II (RII).

## Materials and Methods

### Animals

Four to five week-old female CBA/J mice were obtained from Jackson Laboratory (Bar Harbor, ME, USA) and maintained in micro-isolator cages under specific pathogen free conditions in a BL-2 facility. Cages were housed in a HEPA-filtered animal isolator clean room (Nuaire Inc., Plymouth, MN, USA) and all animal manipulations were performed in class II biological safety cabinets. Virally primed mice were kept physically isolated from all other experimental and stock mice.

### Ethics Statement

This study was carried out in strict accordance with the recommendations in the Guide for the Care and Use of Laboratory Animals of the National Institutes of Health. The protocol was approved by the Stony Brook University Institutional Animal Care and Use Committee (IACUC) (Protocol # 235392-5).

### Virus

Reovirus 1/L was originally obtained from Dr. W. Joklik (Duke University School of Medicine, Durham, NC, USA). Third-passage gradient-purified stocks were obtained by re-cloning and amplifying parental stocks on L-929 fibroblast cells (American Type Culture Collection, Rockville, MD, USA) [5], [6]. Following the purification of new stocks, infectious viral titers were obtained by limiting dilution on L-929 monolayers [5], [6].

### Inoculation Protocol

Animals were lightly anesthetized with an i.p. injection of 100 µl of 18% Ketamine (Vetalar 100 mg/ml; Fort Dodge Laboratories, Inc., Fort Dodge, IA, USA) and 10% Xylazine (Xylazine HCl 20 mg/ml; Phoenix Scientific, Inc., St. Joseph, MO, USA) in sterile injectable grade 0.9% NaCl (Baxter Healthcare Corp., Deerfield, IL, USA) prior to immunization. Animals were inoculated by the intranasal (i.n.) application of 10^7^ pfu of reovirus 1/L in 30 µl (15 µl in each nostril) in 0.9% NaCl. Control animals were inoculated with 30 µl (15 µl in each nostril) 0.9% NaCl. Mice were treated with curcumin (≥94% curcuminoid content) (Sigma-Aldrich, St. Louis, MO, USA) at a concentration of 50 mg/kg in 2% carboxymethylcellulose (CMC) in a total volume of 100 µl delivered by an i.p. injection 5 days prior to infection and daily, thereafter. A curcumin dosage of 50 mg/kg was chosen after a series of initial experiments analyzing histologically the inflammatory infiltrate and fibrotic lesions using curcumin concentrations ranging from 50 mg/kg to 200 mg/kg (data not shown). As controls, either saline or reovirus 1/L-infected mice received a daily i.p. injection of 2% CMC (vehicle). Saline mice treated with 2% CMC (vehicle) did not develop any significant pathology (not shown). Likewises, reovirus 1/L-infected mice treated with 2% CMC (vehicle) did not show any significant improvement in lung pathology (not shown). Since our initial finding indicated that vehicle treatment alone does not modulate key features of reovirus 1/L-ALI/ARDS (inflammation and fibrosis), we compared untreated reovirus 1/L-ALI/ARDS to curcumin-treated reovirus 1/L-ALI/ARDS to minimize stressing the animals unnecessarily. After the indicated time points, animals were sacrificed via carbon dioxide inhalation (Euthanex^TM^ CO_2_ system).

### Titration of infectious virions

Freshly harvested lungs (without the associated lymph nodes) were flash frozen in liquid nitrogen and stored at −80°C until use. Frozen lungs were freeze-thawed three times in 1 ml MEM containing 10% FBS followed by homogenization for 1.5 minutes with a Mini-Beadbeater (Biospec Products, Bartlesville, OK). Lung supernatants were then subjected to centrifugation at 2000 g for 10 minutes at 4°C. Serial dilutions of tissue homogenates (100 µl) in gel-saline were used in a standard plaque assay to determine reovirus 1/L pfu/ml on L-929 cell monolayers as previously described [36].

### Histology

Lungs were inflated *in situ* with 2% paraformaldehyde (Sigma-Aldrich) by intra-tracheal intubation, removed, and suspended in an additional 2% paraformaldehyde for 2 hours at 4°C before being embedded in paraffin. Histological staining was performed on 4-micron sections by McClain Laboratories (Smithtown, NY, USA). Mason's Trichrome and Sirius red staining were used to visualize collagen deposition. With Mason's Trichrome, the nuclei stain a dark red/purple, muscle stains red, and connective tissue including collagen stains blue. With Sirius red, in bright-field microscopy, collagen is red on a yellowish background. Nuclei, if stained, are black but may often be grey or brown. To score lung inflammation and fibrosis, lung samples were screened for the following three histopathological parameters: (a) deposition of extracellular matrix; (b) leukocyte infiltration (interstitial inflammation); and (c) airway obliteration due to granulation tissue formation and/or fibrosis. Each slide was blindly evaluated using low (4X and 10X) and high (20X and 40X) magnification and scored on a scale of 0–3 with 0 as absent (normal); 1 as mild; 2 as moderate; and 3 as severe [5], [6]. The final histology score was based on evaluation of whole lung sections (one section per mouse) and values reported are an average of 18 (reovirus 1/L–ALI/ARDS d9 and d14), 12 (curcumin-treated reovirus 1/L-ALI/ARDS d9) and 28 (reovirus 1/L-ALI/ARDS d14) individual assessments. Images from low (20X) and high (40X) magnification on an Olympus BX40 microscope (Olympus, Melville, NY, USA) were captured with a Polaroid digital microscope camera and edited using Adobe Photoshop 5.0 software.

### Sircol Assay for Collagen Content

Soluble lung collagen was measured in lung homogenates using the Sircol Assay (Biocolor Ltd, UK), following manufacturer's instructions. Briefly, the right lobe of the lung was suspended at 30 mg/tissue/ml in 0.5 M acetic acid and homogenized for 3 min with a Mini-Beadbeater (Biospec Products, Bartlesville, OK, USA). Cellular debris was pelleted by centrifugation and 100 µl of the supernatant was mixed with the Sircol dye and centrifuged. The pellets were dissolved with alkaline reagent, as directed. Absorbance was read at 540 nm. Total soluble collagen was determined using a standard curve. The Sircol Assay is suitable for monitoring collagen produced *in situ* where collagen that is soluble in cold acid/pepsin can be recovered and measured from mammalian soft tissues, cartilages and fluids.

### Flow Cytometric Analysis

Lungs were harvested and digested with 5 ml 1 mg/ml collagenase H (Roche, Nutley, NJ, USA) at 37°C for single cell isolation. Cells were washed three times with PBS containing 5% FCS and 0.05% azide, and resuspended at 10^6^ cells/ml. Cells were stained for surface markers as described previously [5], [6]. The following antibodies (Abs) were purchased from BD Biosciences (San Jose, CA, USA) and used in this analysis: CD4 (GK1.5, L3T4; R-PE-labeled); CD8a (53–6.7, PerCP-labeled); CD11b/Mac-1 (M1/70, integrinα_m_ chain, Mac-1 α chain, APC-labeled); CD19 (1D3, FITC-labeled); Ly6G (RB6-8C5, Gr-1, PerCP-Cy5.5-labeled); and R-PE-conjugated rat anti-mouse Pan-NK cells (DX5). Isotype matched controls were run for each sample (not shown). Flow cytometric analysis was performed using a dual-laser Accuri C6 flow cytometer (BD Accuri, Ann Arbor, MI, USA) and data was analyzed using FlowJo Analysis Software (Tree Star Inc., Ashland, OR, USA).

### Immunofluorescence (IF)

Immunostaining of paraformaldehyde-fixed and paraffin embedded lung sections was performed by IF procedures. Five to eight-micron sequential sections were collected on poly-L-lysine treated slides (Sigma-Aldrich), deparaffinized in xylene and dehydrated in graded alcohol. Tissue sections were then heat treated for 10 minutes with a Target Retrieval Solution (Dako Corp. S1700; Carpinteria, CA, USA) following manufacture's instructions. Tissue sections were permeabilized in 0.2% Triton X-100 in PBS for 45 minutes at room temperature, followed by incubation in 10% BSA for 1 hr. The sections were then incubated overnight at 4°C with a rat anti-tenascin-C (TN-C) Ab (1∶200, Sigma-Aldrich), a mouse anti-α-smooth muscle actin (α-SMA) Ab (1∶400, Sigma-Aldrich), or a goat anti-E-cadherin Ab (1∶200, R & D Systems, Minneapolis, MN, USA). After three washes in PBS, slides were incubated with the corresponding secondary Abs conjugated with photostable Alexa Fluor® dye (1∶1000, Life Technologies Corporation, Grand Island, NY, USA) for 1 hr at room temperature. Slides were treated with Vectashield anti-fade mounting medium with DAPI to stain nuclei (Vector Laboratories) and viewed with a Nikon Eclipse TE2000-S microscopy system. For control incubations, primary Abs was replaced by normal mouse, goat, or rat serum (Vector Laboratories).

### Serum aspartate aminotransferase (AST), alanine aminotransferase (ALT) and alkaline phosphatase (AP) activities

Serum was collected by cardiac puncture at the time of euthanasia and was stored at −80°C until use. Serum AST, ALT and AP activities were measured enzymatically using standardized enzyme assays kits obtained from Stanbio Laboratory (Boerne, TX, USA).

### RNA Preparation and Quantitative RT-PCR (qRT-PCR)

Lung tissue was homogenized in the presence of TRI Reagent® Solution and total RNA was isolated according to the manufacturer's instructions (Applied Biosystems, Life Technologies, Carlsbad, CA, USA). qRT-PCR was carried out in 20 µl total volume containing 100 ng of RNA and 200 nM of each primer using a Power SYBR® Green RNA-to-C_T_™ 1-Step Kit (Applied Biosystems). After reverse transcription for 30 min at 48°C and a starting denaturation for 10 minutes at 95°C, 40 PCR cycles (15 s 95°C and 1 min 60°C) were performed using the ABI StepOne Plus™ Real-Time PCR System® (Applied Biosystems). Oligonucleotide primers used in qRT-PCR are listed in Table 1. Each sample was evaluated in triplicate. Specificity of PCR products was evaluated at the end of each run by melt curve analysis. A non-template control and an endogenous control (GAPDH) were used for relative quantification. All quantitations (threshold cycle [CT] values) were normalized to that of GAPDH to generate ΔCT, and the difference between the ΔCT value of the sample and that of the reference (saline (control)) was calculated as ΔΔCT. The relative level of gene expression was expressed as 2^−ΔΔCT^ [R].

**Table 1:** Primers used for qRT-PCR and RT-PCR.

Gene	Forward (5'–3')	Reverse (5'–3')
Pro-collagen 1	TTCACCTACAGCACGCTTGTG	GATGACTGTCTTGCCCCAAGTT
IL-6	GAGGATACCACTCCCAACAGACC	AAGTGCATCATCGTTGTTCATACA
IL-10	CCCTTTGCTATGGTGTCCTT	TGGTTTCTCTTCCCAAGACC
IFNγ	AGCGGCTGACTGAACTCAGATTGTAG	GTCACAGTTTTCAGCTGTATAGGG
GM-CSF	CACCCGCTCACCCATCAC	TTCTTTGATGGCCTCTACATGCT
MCP-1	GTCTGTGCTGACCCCAAGAAG	TGGTTCGATCCAGGTTTTTA
TGFß1	GACTCTCCACCTGCAAGACCA	GGGACTGGCGAGCCTTAGTT
TGFß-RII	CACGACCCCAAGCTCACCTA	TTGGGAGAAGCGGCATCTT
TN-C	ACGGCTACCACAGAAGCTG	CGCGGCTTATTCCATAGAGTTC
α-SMA	GTCCCAGACATCAGGGAGTAA	TCGGATACTTCAGCGTCAGGA
E-cadherin	CAGGTCTCCTCATGGCTTTGC	CTTCCGAAAAGAAGGCTGTCC
Reovirus M3	TCACAACCCTTCACTCCGTCTG	AAATAATCCGCAGTCTCCAACG
GAPDH	CATGGCCTTCCGTGTTCCTA	GCGGCACGTCAGATCCA

### Western Blot Analysis

For western blotting, lungs were removed and homogenized in 1.5 ml Tissue PE LB^TM^ tissue protein extraction lysis buffer containing a cocktail of protease inhibitors (N-Ethylmaleimide [10 mM], benzamidine [5 mM], leupeptin [50 µg/ml], pepstatin A [5 µg/ml], PMSF [2 mM]) using a Mini-Beadbeater (Geno Technology, St. Louis, MO, USA). Total protein was determined using a modified Bradford protein assay (Sigma-Aldrich). Twenty micrograms of total protein were mixed with 4X sample buffer containing 5% 2-mercaptoethanol, resolved by SDS-PAGE (Ready Gels Tris HCl 10%; Bio-Rad, Hercules, CA, USA), and transferred to PVDF membranes. Membranes were then blocked for 2 hours in 5% non-fat milk in TBST, and incubated overnight at 4°C with either a rat anti-TN-C (Sigma-Aldrich), a mouse monoclonal anti-α-SMA (Sigma-Aldrich), a polyclonal goat anti-E-cadherin (R&D Systems), a rabbit anti-phosph-p65-NFκB, a rabbit anti-phosph-p38 or a rabbit anti-ß actin (Cell Signaling Technology, Danvers, MA, USA) Ab. The membranes were then washed 3X and incubated with the appropriate secondary Abs conjugated to horseradish peroxidase (Thermo-Scientific) for one and a half hours at room temperature. Western analysis using an anti-ß actin Ab (Santa Cruz Biotechnology, Inc.) was performed to demonstrate equal loading. Immunostained bands were visualized with SuperSignal® West Pico Chemiluminescent Substrate kit (Thermo-Scientific) followed by exposure to HyBlot CL^TM^ Autoradiography Film (Denville Scientific Inc, Metuchen, NJ, USA). Band intensities on scanned gels were analyzed using the public domain National Institutes of Health ImageJ program.

### Statistical analysis

Statistical analyses were performed by using an ANOVA with treatment and time of study included as factors. When appropriate, comparisons between two variables were performed using a Student's *t* test. A *p* value less than 0.05 was considered significant.

## Results

### Administration of Curcumin protects CBA/J mice from the Development of ALI/ARDS in Response to reovirus 1/L

**Figure 1:**
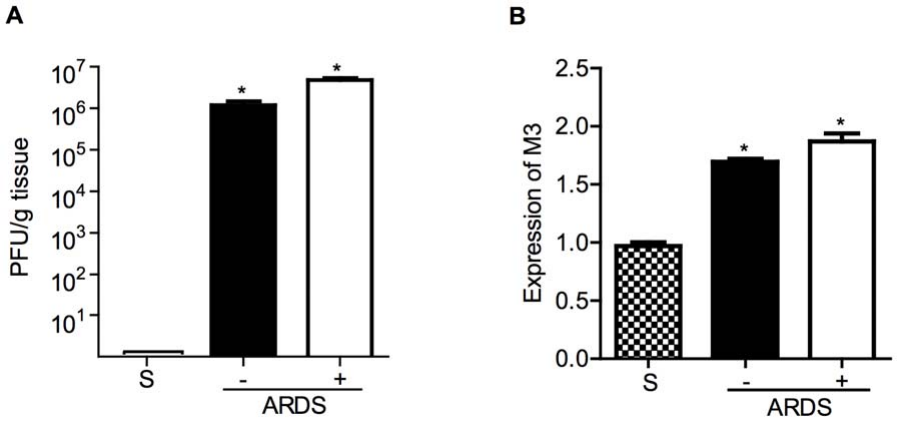
Administration of curcumin does not effect reovirus 1/L clearance or replication in vivo. CBA/J mice were inoculated i.n. with 10^7^ pfu reovirus 1/L and were either untreated (−) or treated (+) with 50 mg/kg curcumin by i.p. injection beginning 5 days prior to infection and daily, thereafter. Whole lungs were removed on day 9 post-infection. (A) Lungs were individually homogenized and pfu were determined by a standard plaque assay. Results are expressed as the average number of pfu per g lung tissue. No plaques were detected in saline inoculated mice. Histograms are the mean +/− S.D. of one experiment with three mice per time point. *p<0.05 versus saline (control). (B) RNA was prepared from whole lung tissue from saline (S) or from untreated (−, solid bars) or curcumin-treated (+, open bars) reovirus 1/L-ALI/ARDS and relative expression of reovirus 1 L M3 gene was assessed by qRT-PCR. Histograms are the mean +/− S.D. of one experiment with three mice per time point. *p<0.05 versus saline (control).

**Figure 2:**
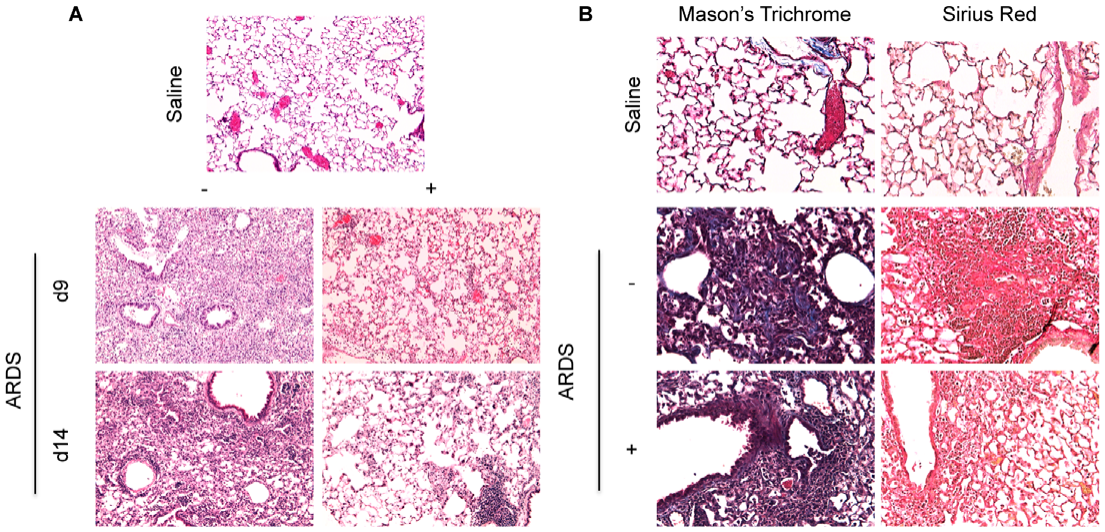
Administration of curcumin protects CBA/J mice from the development of ALI/ARDS in response to reovirus 1/L. CBA/J mice were inoculated i.n. with 10^7^ pfu reovirus 1/L and were either untreated (−) or treated (+) with 50 mg/kg curcumin by i.p. injection beginning 5 days prior to infection and daily, thereafter. Whole lungs were removed at the indicated time points, fixed in 2% paraformaldehyde and embedded in paraffin. Staining was performed on 4-micron sections. (A) H&E Staining on days 9 and 14; (B) Mason's Trichrome and Sirius Red Staining on day 14. Objective magnification: 10X (A) and 20X (B). Representative of at least four independent experiments evaluating 3 mice per time point.

In order to determine the efficacy of treatment with curcumin on the development of either ARDS associated DAD or fibrosis, CBA/J mice were inoculated i.n. with 10^7^ pfu reovirus 1/L and were either untreated or treated with 50 mg/kg curcumin by i.p. injection 5 days prior to infection and daily, thereafter. We first evaluated whether daily curcumin treatment of mice *in vivo* affected the ability of reovirus 1/L to infect or replicate in reovirus 1/L-inoculated mice (Fig. 1). We demonstrate on day 9 post-reovirus 1/L-inoculation that both the number of reovirus 1/L infectious virions (Fig. 1A) as well as the ability of reovirus 1/L to replicate *in vivo* (Fig. 1B) are not affected by curcumin treatment. Thus, we demonstrate that the mechanism of action of curcumin in this model is not due to affects on replication or clearance of reovirus 1/L from infected lungs. To determine the extent of inflammation and fibrosis, paraffin-embedded lung sections were stained with H&E, Sirius Red, or Masson's Trichrome (Fig. 2). Inflammation (as evidenced by DAD) was evaluated on day 9 and fibrosis was evaluated on day 14. All untreated, reovirus 1/L-ALI/ARDS mice developed a viral pneumonia including symptoms and histological features of ARDS, as previously described [5], [6]. Specifically, reovirus 1/L-ALI/ARDS mice developed a significant diffuse cellular infiltrate leading to DAD and severe pneumonia (Fig. 2A; day 9, grade: 2.3 +/−0.46). Accompanying this DAD are edema, capillary dilation and hemorrhage, and the formation of hyaline membranes (not shown). Those mice that survive the acute phase of the disease develop interstitial fibrosis and intra-alveolar fibrosis (Fig. 2A; day 14, grade: 2.1 +/−0.50). In contrast, curcumin-treated reovirus 1/L-ALI/ARDS mice developed significantly less of a cellular infiltrate with a patchy distribution and formation of condensed focal lymphocytic accumulations (Fig. 2A; day 9, grade: 1.31 +/−0.44; p<0.05 versus reovirus 1/L-ALI/ARDS). In addition, the intra-alveolar and interstitial fibrosis associated with the late phase of reovirus 1/L-ALI/ARDS was also significantly inhibited by treatment with curcumin (Fig. 2A; day 14, grade: 1.31 +/−0.49; p<0.05 versus reovirus 1/L-ALI/ARDS). To more clearly demonstrate the presence of fibrotic lesions histologically, tissue sections were stained with Mason's Trichrome or Sirius red on day 14 post-infection (Fig. 2B). In normal or saline (control) lung sections, Sirius red or Mason's Trichrome staining for collagen is evident only within the walls of the bronchioles and arterioles, which contain connective tissue including collagen, while the lung alveolar airspaces are not stained (Fig. 2B). As can be observed, significant staining for collagen via either Mason's Trichrome (blue) or Sirius red (bright red) is evident in reovirus 1/L-ALI/ARDS mice, which is dramatically reduced in curcumin-treated reovirus 1/L-ALI/ARDS mice (Fig. 2B).

**Figure 3:**
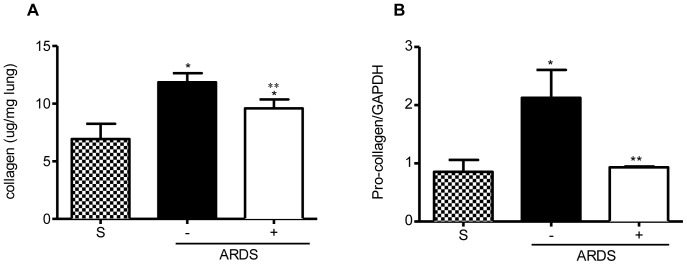
Administration of curcumin reduces collagen deposition in reovirus 1/L-ALI/ARDS. CBA/J mice were inoculated i.n. with 10^7^ pfu reovirus 1/L and were either untreated (−) or treated (+) with 50 mg/kg curcumin by i.p. injection beginning 5 days prior to infection and daily, thereafter. (A) Whole lungs were removed from saline (S), untreated (−, solid bars) or curcumin-treated (+, open bars) reovirus 1/L-ALI/ARDS on day 14 post-infection and soluble lung collagen was measured using the Sircol Assay as per manufacturer's instructions; (B) RNA was prepared from whole lung tissue from saline (S) or from untreated (-, solid bars) or curcumin-treated (+, open bars) reovirus 1/L-ALI/ARDS on day 14 post-infection and relative expression of pro-collagen 1 was assessed by qRT-PCR. Histograms are the mean +/− S.D. of two experiments with three mice per time point. *p<0.05 versus saline (control), **p<0.05 versus reovirus 1/L-ALI/ARDS.

To support the histological evaluation of fibrosis, the Sircol™ Collagen Assay demonstrated significant collagen accumulation in the lungs at day 14-post infection in untreated, reovirus 1/L-ALI/ARDS mice as compared to saline (control) lungs (approximately a 2-fold increase) (Fig. 3A). In contrast, the amount of collagen accumulation in the lungs of curcumin-treated reovirus 1/L-ALI/ARDS mice is significantly reduced versus the reovirus 1/L-ALI/ARDS mice, even though they still accumulate more collagen than saline (control) mice (Fig. 3A). As an additional measure of collagen accumulation, mRNA for pro-collagen I was measured on day 14 post-infection (Fig. 3B). Reovirus 1/L-ALI/ARDS mice expressed a significantly higher level of pro-collagen I mRNA as compared to saline (control) mice, which was significantly reduced in curcumin-treated, reovirus 1/L-ALI/ARDS mice (Fig. 3B).

**Figure 4:**
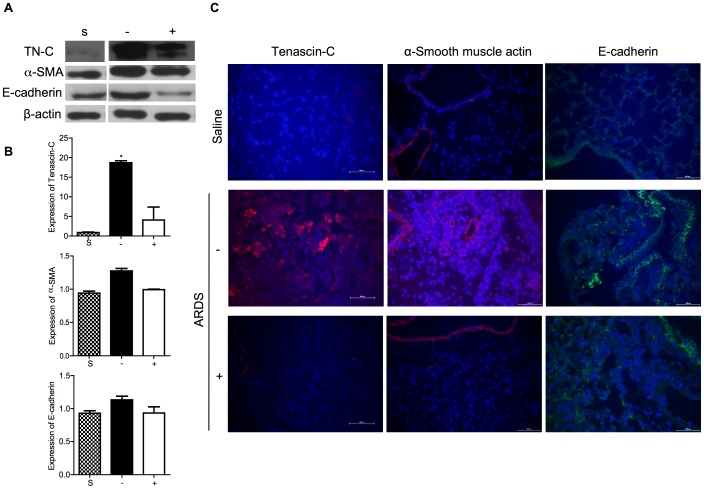
Administration of curcumin reduces expression of the myofibroblast cell phenotype in reovirus 1/L-ALI/ARDS. CBA/J mice were inoculated i.n. with 10^7^ pfu reovirus 1/L and were either untreated (−) or treated (+) with 50 mg/kg curcumin by i.p. injection beginning 5 days prior to infection and daily, thereafter. (A) Western analysis from whole lung lysates for protein expression of TN-C, α-SMA, and E-cadherin from either saline (S), untreated (−), or curcumin-treated (+) reovirus 1/L-ALI/ARDS mice on day 14 post-inoculation. ß-actin expression demonstrated equal loading. Representative of three mice per time point; (B) RNA was prepared from whole lung tissue on day 14 post-inoculation and the relative expression of TN-C, α-SMA, and E-cadherin was assessed by qRT-PCR from untreated (−, solid bars) or treated (+, open bars) reovirus 1/L-ALI/ARDS mice. Saline inoculated mice were used as controls (S, stippled bars). Histograms are the mean +/− S.D. of three mice per time point. *p<0.05 versus saline (control). (C) Lungs of saline, untreated (−) or curcumin-treated (+) reovirus 1/L-ALI/ARDS mice on day 14 post-inoculation were paraffin embedded and stained with an Abs to TN-C (red), α-SMA (red), or E-cadherin (green). Representative of two independent experiments.

To provide further evidence for the anti-fibrogenic action of curcumin in reovirus 1/L-ALI/ARDS, we analyzed pulmonary expression of TN-C, an extracellular matrix glycoprotein that is highly expressed in adult lung parenchyma following acute lung injury [37]–[40] and pulmonary levels of α-SMA and E-cadherin, two well-established markers of epithelial to mesenchymal transition (Fig. 4). As evaluated by western, TN-C is minimally expressed in adult saline (control) lung tissue (Fig. 4A). In untreated, reovirus 1/L-ALI/ARDS lung tissue, the 190-kDa isoform of TN-C is highly expressed on day 14 post-infection. However, in the presence of curcumin, induction of TN-C is significantly reduced on day 14 post-reovirus 1/L inoculation (Fig. 4A). Western analysis data for TN-C expression was confirmed by mRNA expression via qRT-PCR (Fig. 4B). By IF, TN-C expression is minimal in saline (control) lung (Fig. 4C). However, TN-C is highly induced in untreated, reovirus 1/L-ALI/ARDS lungs on day 14 especially visible in areas of lung injury (Fig. 4C, red). In curcumin-treated reovirus 1/L-ALI/ARDS mice, the presence of TN-C was significantly reduced (Fig. 4C). Similarly by IF, expression of α-SMA was negligible in saline (control) mice being observed only within the walls of the bronchioles and vasculature (Fig. 4C, red). However, a marked induction of α-SMA expression was observed in reovirus 1/L-ALI/ARDS, in agreement with the increased deposition of fibrotic tissue (Fig. 4C, red). In contrast, curcumin-treated reovirus 1/L-ALI/ARDS mice demonstrated a marked reduction in α-SMA expression (Fig. 4C). The expression of α-SMA was confirmed by protein expression via western analysis and by mRNA expression via qRT-PCR (Fig. 4A and B). In saline (control) lungs, E-cadherin is clearly expressed by the alveolar epithelial cells (Fig. 4C, green). However, during post injury, lung remodeling is associated with increased E-cadherin expression, which occurs simultaneously with the peak of cellular proliferation and repair and E-cadherin expression is reduced in curcumin-treated reovirus 1/L-ALI/ARDS (Fig. 4C, green). The expression of E-cadherin was confirmed by protein expression via western analysis and by mRNA expression via qRT-PCR (Fig. 4A and B).

### Administration of Curcumin suppresses serum liver enzyme levels in reovirus 1/L-ALI/ARDS

**Figure 5:**
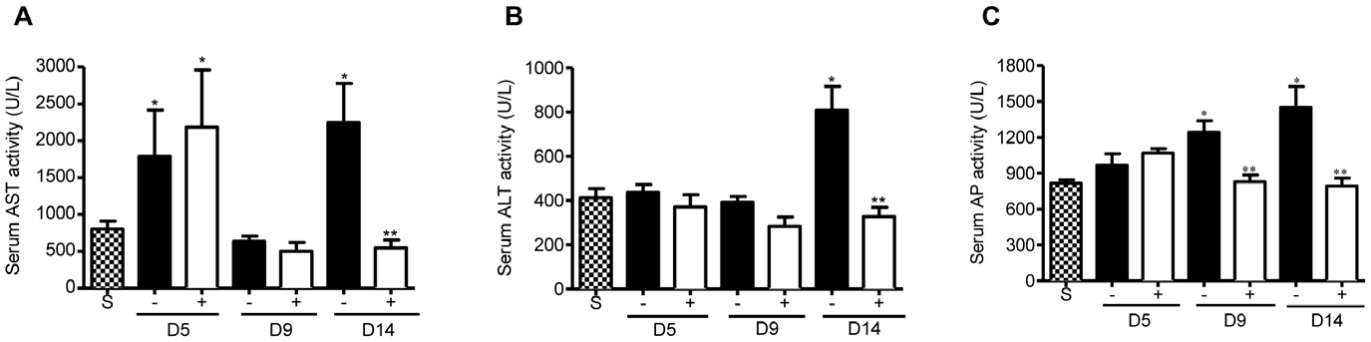
Administration of curcumin reduces expression of the key liver enzymes in the serum during reovirus 1/L-ALI/ARDS. CBA/J mice were inoculated i.n. with 10^7^ pfu reovirus 1/L and were either untreated (−, solid bars) or treated (+, open bars) with 50 mg/kg curcumin by i.p. injection beginning 5 days prior to infection and daily, thereafter. Saline inoculated mice were used as controls (S, stippled bars). Serum was collected at the indicated time points. (A) Serum AST activity; (B) Serum ALT activity; (C) Serum AP activity. Histograms are the mean +/− S.D. of two experiments with three mice per time point. *p<0.05 versus saline (control), **p<0.05 versus reovirus 1/L-ALI/ARDS.

We also analyzed changes in liver enzymes to detect hepatic dysfunction as evidence of a systemic response after reovirus 1/L-ALI/ARDS and whether treatment with curcumin affected potential hepatic dysfunction (Fig. 5). Serum enzyme analysis indicated an early significant increase in serum AST activity over saline (control) mice on day 5 post-inoculation in either untreated or curcumin-treated reovirus 1/L-ALI/ARDS mice which was reduced to control levels on day 9 post-inoculation (Fig. 5A). However, on day 14 a significant increase in serum AST activity was observed in untreated, reovirus 1/L-ALI/ARDS which was reduced to control levels in curcumin-treated, reovirus 1/L-ALI/ARDS mice (Fig. 5A). Both serum ALT activity (Fig. 5B) on day 14 and AP activity (Fig. 5C) on both days 9 and 14 post-inoculation were significantly increased over saline (control) levels in reovirus 1/L-ALI/ARDS mice that was reduced to control levels in curcumin-treated, reovirus 1/L-ALI/ARDS mice.

### Curcumin modulates the Phenotype of the Inflammatory Cellular Infiltrate in Reovirus 1/L-ALI/ARDS Mice

**Figure 6:**
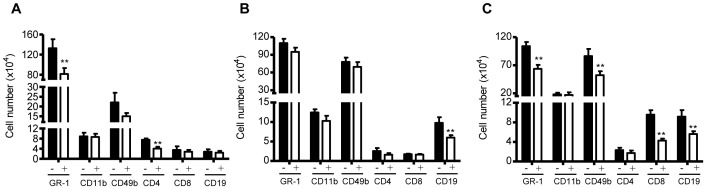
Administration of curcumin modulates the inflammatory cell infiltrate during reovirus 1/L-ALI/ARDS. CBA/J mice were inoculated i.n. with 10^7^ pfu reovirus 1/L and were either untreated (−, solid bars) or treated (+, open bars) with 50 mg/kg curcumin by i.p. injection beginning 5 days prior to infection and daily, thereafter. Infiltrating cells were recovered from the lungs and analyzed for the expression of the indicated cell surface phenotype markers, as described. (A) Day 5; (B) Day 9; (C) Day 14. Histograms are the mean +/− S.D. of two experiments with three mice per time point. **p<0.05 versus reovirus 1/L-ALI/ARDS.

To determine the percentage over time of different leukocyte subsets present in the inflammatory infiltrate, infiltrating cells from whole lung preparations were obtained on days 5, 9, and 14 post reovirus 1/L inoculation and analyzed by flow cytometry using monoclonal Abs specific for T cell subsets (CD4, CD8), B cells (CD19), Macrophages (CD11b), neutrophils (GR-1), and NK cells (CD49B (pan-NK)) (Fig. 6). In saline or saline + curcumin-treated (control) mice, approximately 0.77+/−0.20×10^6^ cells were recovered from the interstitial spaces with macrophages and PMNs being the predominate cells present (Table 2; not shown). A significant increase in the total number of inflammatory cells was recovered from either untreated reovirus 1/L-ALI/ARDS or curcumin-treated reovirus 1/L-ALI/ARDS mice on days 5, 9, or 14 as compared to saline or saline + curcumin-treated (control) mice (Table 2). Further, the number of recovered cells on days 5 and 14 from curcumin-treated reovirus 1/L-ALI/ARDS mice was significantly less than the number recovered from untreated reovirus 1/L-ALI/ARDS (Table 2). In regards to phenotype, a significant decrease in the number of PMNs (GR1^+^) and CD4^+^ cells on day 5 and CD19^+^ B cells on day 9 was observed in curcumin-treated reovirus 1/L-ALI/ARDS mice versus untreated reovirus 1/L-ALI/ARDS mice (Fig. 6A and B). On day 14, as compared to reovirus 1/L-ALI/ARDS, a significant decrease in PMNs, NK cells, CD8+ T cells and CD19+ B cells was observed in curcumin-treated reovirus 1/L-ALI/ARDS mice (Fig. 6C).

**Table 2:** Number of cells recovered from untreated and curcumin-treated reovirus 1/L-ALI/ARDS^a^

Group	Day 5^b^	Day 9	Day 14
Reovirus 1/L-ALI/ARDS	1.76+/−0.61*	2.14+/−0.42*	2.30+/−0.65*
Curcumin-treated Reovirus 1/L-ALI/ARDS	1.15+/−0.34*, **	1.84+/−0.40*	1.45+/−0.46*, **

a CBA/J mice were inoculated i.n. with 10^7^ pfu reovirus 1/L and were either untreated or treated with 50 mg/kg curcumin by i.p. injection at 5 days prior to infection and daily, thereafter. At the indicated time points, infiltrating cells were recovered from the lungs. In saline or saline + curcumin-treated mice (controls), approximately 0.77+/−0.20×10^6^ cells were recovered from the interstitial spaces.

b Mean number of cells ×10^6^ +/−S.D. of two experiments with four mice per time point.

* p<0.05 versus saline

** p<0.05 versus reovirus 1/L-ALI/ARDS

**Figure 7:**
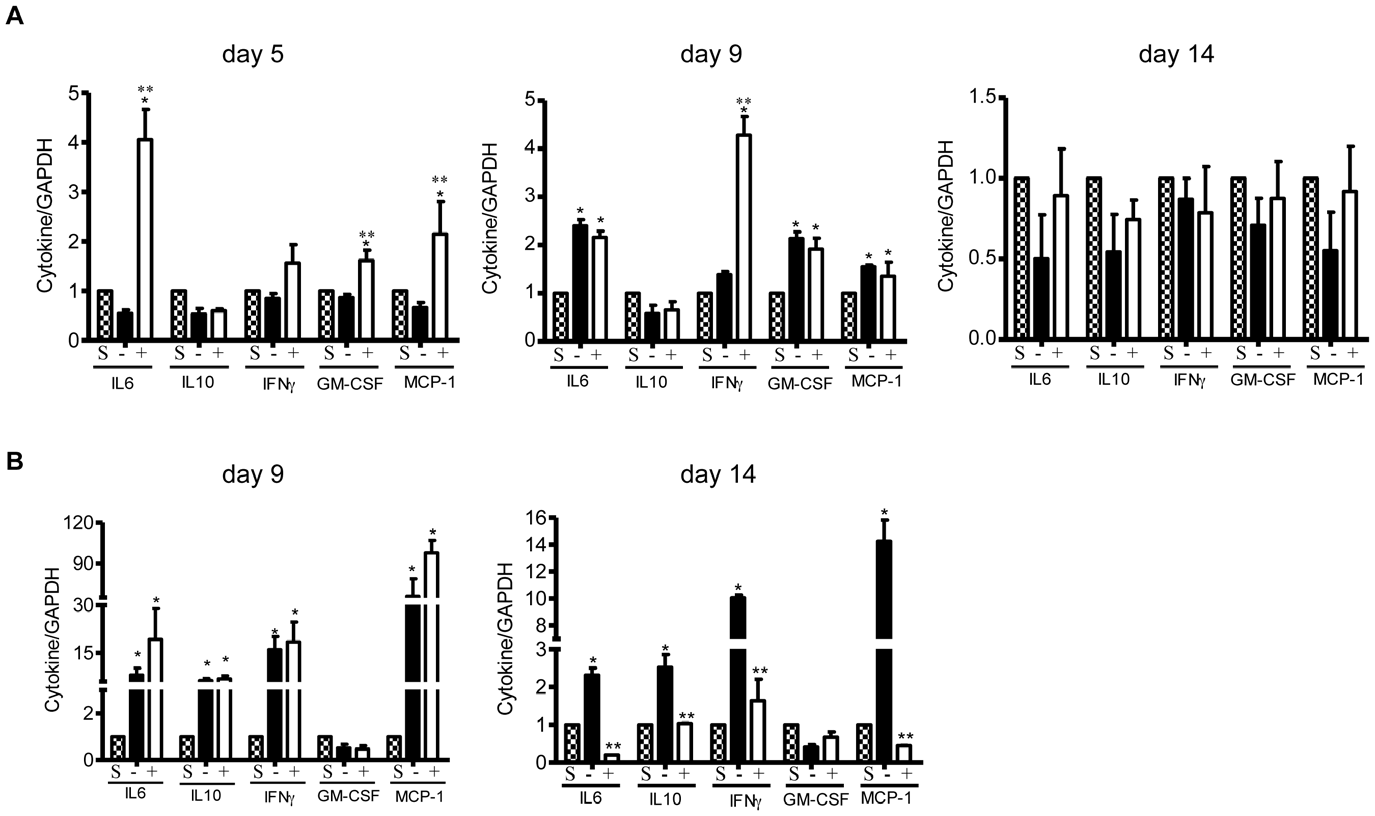
Administration of curcumin modulates key cytokine/chemokine expression during reovirus 1/L-ALI/ARDS. CBA/J mice were inoculated i.n. with 10^7^ pfu reovirus 1/L and were either untreated (−, solid bars) or treated (+, open bars) with 50 mg/kg curcumin by i.p. injection beginning 5 days prior to infection and daily, thereafter. At the indicated time points, RNA was prepared from (A) the infiltrating cells; or (B) whole lung tissue and relative expression of key cytokines/chemokines was assessed by qRT-PCR. Saline inoculated mice were used as controls (S, stippled bars). Histograms are the mean +/− S.D. of two experiments with three mice per time point. *p<0.05 versus saline (control), **p<0.05 versus reovirus 1/L-ALI/ARDS.

To determine whether the cytokine/chemokine expression profile of the infiltrating cells from reovirus 1/L-ALI/ARDS mice was modulated during curcumin treatment, RNA from infiltrating cells on days 5, 9, and 14 was evaluated for expression of key cytokines/chemokines including IL-6, IL-10, IFNγ, G/M-CSF, and MCP-1 via qRT-PCR (Fig. 7). Interestingly, an early significant increase in the expression of IL-6, G/M-CSF and MCP-1 was observed on day 5 in curcumin-treated reovirus 1/L-ALI/ARDS as compared to untreated reovirus 1/L-ALI/ARDS mice or saline (control) mice (Fig. 7A). However, by day 9 post-inoculation, the expression of IL-6, G/M-CSF and MCP-1 was significantly higher in both untreated versus curcumin treated reovirus 1/l-ALI/ARDS mice as compared to saline (control) mice (Fig. 7A). In addition, on day 9 the expression of IFNγ, was significantly increased in curcumin-treated reovirus 1/L-ALI/ARDS mice as compared to untreated reovirus 1/L-ALI/ARDS mice and saline (control) mice (Fig. 7A). By day 14 in either untreated or curcumin-treated reovirus 1/L-ALI/ARDS, the expression of IL-6, IL-10, IFNγ, GM-CSF and MCP-1 were similar to that of saline (control) mice (Fig. 7A). In addition, cytokine/chemokine mRNA expression from whole lung tissue (including the infiltrating cells) was also evaluated on days 9 and 14 post-inoculation by qRT-PCR. Expression of IL-6, IL-10, IFNγ, and MCP-1 was significantly increased over saline (control) in both untreated and curcumin-treated reovirus 1/L-ALI/ARDS on day 9 post-inoculation (Fig. 7B). However, at day 14 post-inoculation, the expression of IL-6, IL-10, IFNγ, and MCP-1 in reovirus 1/L-ALI/ARDS mice was still significantly increased over saline (control) mice, which was significantly inhibited in curcumin-treated reovirus 1/L-ALI/ARDS mice (Fig. 7B).

### Curcumin differentially modulates the TGFß1 NFκB, and the p38 signaling pathways

**Figure 8:**
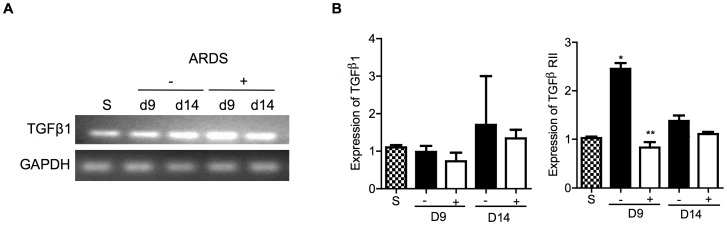
Administration of curcumin modulates TGFß RII expression during reovirus 1/L-ALI/ARDS. CBA/J mice were inoculated i.n. with 10^7^ pfu reovirus 1/L and were either untreated (−, solid bars) or treated (+, open bars) with 50 mg/kg curcumin by i.p. injection beginning 5 days prior to infection and daily, thereafter. At the indicated time points, RNA was prepared from whole lung tissue and the relative expression of (A) TGFß1 was assessed by RT-PCR; (B) TGFß1 and TGFß RII mRNA expression was also assessed by qRT-PCR. Saline inoculated mice were used as controls (S, stippled bars). Histograms are the mean +/−S.D. of two experiments with three mice per time point. *p<0.05 versus saline (control), **p<0.05 versus reovirus 1/L-ALI/ARDS.

**Figure 9:**
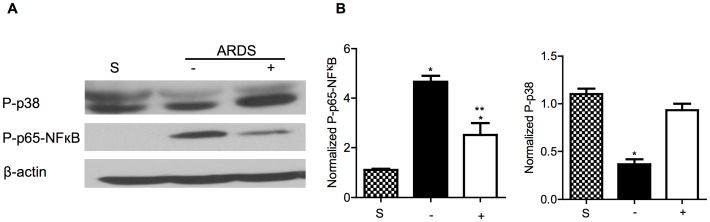
Administration of curcumin modulates the phosphorylation status of the p65 subunit of NFkB and p38 MAPK. CBA/J mice were inoculated i.n. with 10^7^ pfu reovirus 1/L and were either untreated (−) or treated (+) with 50 mg/kg curcumin by i.p. injection beginning 5 days prior to infection and daily, thereafter. (A) On day 9 post-inoculation, protein was extracted from whole lung tissue from untreated (−) or curcumin-treated (+) reovirus 1/L-ALI/ARDS mice and analyzed for the expression of phosphorylated p38 (P-p38) and phosphorylated p65-NFκB (P-p65-NFκB) by western analysis. Saline inoculated mice were used as controls (S). Analysis of ß actin was performed to demonstrate equal loading. Representative of two experiments with three mice per time point. (B) Relative expression of P-p38 and P-p65-NFκB was determined by comparing their expression to that of ß-actin. Histograms represent densitometric data from the mean +/− SD autoradiogram signals from three mice for the saline, untreated (−, solid) or curcumin-treated (+, open) reovirus 1/L-ALI/ARDS*p<0.05 versus saline (control), **p<.0.05 versus reovirus 1/L-ALI/ARDS.

Three key pathways involved in the pathogenesis of ALI/ARDS, TGFß1, NFκB, and p38 were investigated in reovirus 1/L-ALI/ARDS. RNA from whole lung tissue on days 9 and 14 post inoculation was evaluated for expression of TGFß1 and TGFß RII by RT-PCR or qRT-PCR (Fig. 8) and protein extracts from whole lung tissue were evaluated for expression of the phosphorylated form of the p65 subunit of NFκB or the phosphorylated form of p38, an additional key molecule in the TGFß signaling pathway (Fig. 9). While the expression of TGFß1 was not modulated by curcumin on either day 9 or day 14 in reovirus 1/L-ALI/ARDS (Fig. 8A and B), TGFß RII, which is required for TGFß signaling, was significantly reduced on day 9 in the presence of curcumin (Fig. 8B). Curcumin also demonstrated differential effects on expression of the phosphorylated (P) forms of p38 and the p65 subunit of NFκB (Fig. 9). Treatment of reovirus 1/L-ALI/ARDS with curcumin resulted in a significant increase in P-p38 evident on day 9 post-inoculation as compared to untreated reovirus1/L-ALI/ARDS (Fig. 9A and B). However, treatment of reovirus 1/L-ALI/ARDS with curcumin resulted in a significant reduction of P-p65 NFκB as compared to untreated reovirus1/L-ALI/ARDS (Fig. 9A and B).

## Discussion

This study was conducted in a mouse model of infection (reovirus 1/L)-induced *acute viral pneumonia*, which leads to ARDS, the most severe form of ALI. Reovirus 1/L-ALI/ARDS mice demonstrated severely damaged lungs as evidenced by DAD followed by infiltration of the interstitium and alveolar spaces with fibroblasts with excessive collagen deposition [5]–[8]. However, in reovirus 1/L-ALI/ARDS undergoing treatment with curcumin in a pre-clinical model, these inflammatory and fibrotic changes were significantly reduced. Key markers of fibrosis including expression of α-SMA, TN-C and E-cadherin were modified by curcumin. Curcumin also modulated the expression of key chemokines/cytokines implicated in the development of ALI/ARDS including IL-6, IL-10, IFNγ, and MCP-1 from both the inflammatory infiltrate and from whole lung tissue potentially through the modulation of NFκB. While the expression of TGFß1 was not modulated by curcumin, TGFß RII, which is required for TGFß signaling, was significantly reduced. In reovirus 1//L-ALI/ARDS, we also observed dramatic increases in systemic levels of liver enzymes including AST, ALT, and AP especially on day 14 when significant fibrotic lesions are observed. This is similar to many patients with ARDS who die not from hypoxemia but from multiple organ failure [41], [42]. However, curcumin-treated reovirus 1/L-ALI/ARDS mice demonstrated a significant decrease is all three systemic indicators suggesting that curcumin prevents not only the inflammatory reaction but also the later systemic reactions due to continued lung fibrosis and hypoxemia. We also demonstrate in reovirus 1/L-ALI/ARDS an increase in expression of ãSMA and TN-C especially visible in areas of lung injury in agreement with the increased deposition of fibrotic tissue. In contrast, reovirus 1/L-ALI/ARDS mice treated with curcumin demonstrated a marked reduction in α-SMA and TN-C. These data confirm that curcumin induces anti-fibrogenic actions, related at least in part to a lower accumulation of myofibroblasts during the course of the fibrogenic process. Our results also suggest that the curcumin further manifests its effects through the induction of the p38 MAPK, a subfamily of serine-threonine protein kinases, having pro-apoptotic effects in response to extracellular stimuli [43]. Since the effects of curcumin are likely pleotropic and complex, determining the downstream effectors of curcumin will be crucial in analyzing the full potential value of curcumin as a potential therapeutic.

In this report, we demonstrate a differential effect of curcumin on the inflammatory infiltrate versus whole lung tissue, which would include an inflammatory response associated with resident epithelial, endothelial and/or fibroblast cells. We demonstrate from the inflammatory infiltrate an early (day 5) and significant rise in IL-6, G/M-CSF, and MCP-1 in curcumin-treated reovirus 1/L-ALI/ARDS which is not mirrored by untreated reovirus 1/L-ALI/ARDS until day 9. In addition, on day 9, IFNγ expression from the inflammatory infiltrate in curcumin-treated reovirus 1/L-ALI/ARDS is significantly higher than in untreated reovirus 1/L-ALI/ARDS. However, by day 14, the inflammatory infiltrate from either reovirus 1/L-ALI/ARDS or curcumin-treated reovirus 1/L-ALI/ARDS no longer significantly expresses any of these key cytokines/chemokines. However, when evaluating whole lung mRNA for cytokine/chemokine expression, it is evident that resident pulmonary alveolar epithelial, endothelial, and fibroblast cells contribute to the overall cytokine/chemokine storm especially as evident on day 14. While curcumin down regulates the expression IL-6, IL-10, IFNγ and MCP-1 in curcumin-treated reovirus 1/L-ALI/ARDS on day 14, untreated reovirus 1/L-ALI/ARDS express high levels of these cytokines/chemokines especially, IFNγ and MCP-1. These results suggest a consistent and prolonged inflammatory response most likely initiating from resident pulmonary alveolar epithelial cells and fibroblasts in untreated reovirus 1/L-ALI/ARDS, contributed to the pathology associated with ALI/ARDS. We have previously demonstrated a role for IFNγ in the pathophysiology of reovirus 1/L pulmonary pathologies and suggest that prolonged and high levels of IFNγ are associated with more severe pulmonary pathologies [7], [8]. The reduced pathology associated with curcumin treatment and the reduced expression of IFNγ by curcumin would support this hypothesis. We have also demonstrated the potential effect of curcumin on NFκB activation by a decrease in the levels of the P-p65. This result is clearly consistent with the decreased expression of IFNγ and MCP-1, which is observed in curcumin-treated reovirus 1/L-ALI/ARDS. The literature clearly links canonical activation of NFκB in cells to the pathogenesis of inflammatory diseases 44–48. NFκB is activated in the lungs of patients with ALI [49], [50] and persistent elevation of NFκB in patients with sepsis-induced ALI is associated with poor outcome [51], [52]. Curcumin has been demonstrated to modulate NFκB activation in cell systems as well as *in vivo* model systems [16], [18], [53]–[57]. As an anti-inflammatory agent in association with lung diseases curcumin inhibited pro-inflammatory cytokines including IL-8, IL-1, and TNF through the inhibition of the transcription factors NFκB and AP-1 [26], [28]. However, although NFκB plays a major role in regulating expression of cytokines/chemokines, the effects of curcumin in reovirus 1/L-ALI/ARDS cannot solely be attributed to its effect on NFκB since glucocorticoids also inhibit NFκB but do not suppress the development, progression, and mortality associated with reovirus1/L-ALI/ARDS [7], [8].

TGFß and its signaling pathways have also been implicated in lung fibrosis [58]–[60] and TGFß over expression is associated with poorer prognosis in ARDS [61]–[63] as well as in our model [5], [6]. While curcumin has been demonstrated to reduce collagen in experimental pulmonary fibrosis induced by cyclophosphamide, whole-body irradiation, and bleomycin, little mechanistic studies have been performed [20], [22]–[27], [64], [65]. Here, we demonstrate in reovirus 1/L-ALI/ARDS that while treatment with curcumin did not inhibit the expression of TGFß1 itself, expression of TGFß RII was significantly reduced especially on day 9 post-induction where the transition between inflammation and fibrosis is most evident. Although the precise mechanism remains unclear, in other pulmonary fibrotic models including in bleomycin-induced fibrosis [22], [25], [66]–[69], in SiO2-induced fibrosis [70], and in amiodarone-induced fibrosis [23], curcumin has been shown to reduce either TGFß protein or mRNA expression. In amiodarone-induced fibrosis, this reduction in TGFß is potentially through a decrease in c-Jun expression [23]. Aberrant activation of TGFß signaling has also been implicated in scleroderma fibrosis [20], [21], [71], [72] and in keloid, a fibrotic disease characterized by abnormal accumulation of ECM in the dermis via reduction in TGFß/p-SMAD-2 [73]. Over expression of TGFß has also been implicated in fibrotic kidney disease and curcumin inhibited TGFß signaling and reduced TGFß induced increases in PAI-1, TGFß1, collagen, and fibronectin through a reduction in both TGFß RII and phosphorylation of Smad2/3 in renal cells [74], [75]. In hepatic fibrosis curcumin activated PPARγ which contributed to apoptosis and a reduction in extracellular matrix expression in hepatic stellate cells through the interruption of TGFß signaling and suppression of CTGF [76]–[78].

There is convincing evidence in patients that increased lung epithelial apoptosis is associated with poor outcomes in ALI/ARDS [51], [79]–[81]. Pathways involving both the p38 MAP or Akt kinases are important in PMNs and macrophages, the major infiltrating cells in ALI/ARDS [49], [50], [82]. Diminished translocation of NFκB and phosphorylation of Akt but not p38 in PMNs of patients with sepsis-induced ALI was associated with improved survival [49]. It has also been demonstrated that activation of p38 MAPK or an increase in caspase-3 activity appears to contribute to the proapoptotic effect of human neutrophil apoptosis by curcumin [83]. Curcumin has been shown to modulate apoptosis in a number of different cell types. In scleroderma, it was shown that scleroderma lung fibroblasts, which are deficient in PKCε, are sensitive to curcumin-induced apoptosis, whereas normal lung fibroblasts are insensitive to curcumin [20], [21]. The importance of PKCε was demonstrated by the observations that increasing PKCε expression in scleroderma lung fibroblasts provides protection against curcumin and decreasing PKCε expression or activity in normal lung fibroblasts causes the cells to become sensitive to curcumin [20], [21]. In pleural mesothelioma cells, curcumin was shown to activate p38 MAPK, caspases 9 and 3, caused elevated levels of proapoptotic proteins Bax, stimulated PARP cleavage, and induced apoptosis [84]. Curcumin has also been shown to attenuate gefitinib-induced cell proliferation and enhances apoptosis through altering p38 MAPK activation in intestinal epithelia cell [85]. In the reovirus 1/L-ALI/ARDS model, we have clearly demonstrated a role for apoptosis in reovirus 1/L-ALI/ARDS [9]. Although we demonstrated both apoptosis of the alveolar epithelium via TUNEL analysis and the up regulation of Fas and FasL *in situ* in alveolar epithelium and in cells of the inflammatory infiltrate, modulation of reovirus 1/L-ALI/ARDS was not linked to the inhibition of the Fas/FasL pathway suggesting the involvement of additional apoptosis pathways [9]. Here, we demonstrate in curcumin-treated reovirus 1/L-ALI/ARDS, an increase in P-p38, which has been linked to enhanced PMN apoptosis. While we demonstrated a significant decrease in the total number of infiltrating cells and a decrease in the number of PMN on day 14 in curcumin-treated reovirus 1/L-ALI/ARDS, whether this decrease is reflected in an increase in apoptosis is yet to be determined.

In conclusion, our model provides a very relevant model for infection (viral)-induced ALI/ARDS and for deciphering the mechanism(s) of action of agents that show promising efficacy against ALI/ARDS such as the potent anti-inflammatory agent, curcumin. Our data strongly support the hypothesis in a pre-clinical model that treatment of reovirus 1/L-ALI/ARDS with curcumin inhibits the host inflammatory response potentially through the modulation of cytokine/chemokine expression through the NFκB pathway as well as the host fibrotic response during the regeneration phase of the disease through modulation of the TGFß pathway. Our results also suggest that curcumin may modulate apoptosis pathways mediated through the p38 MAPK pathway. Thus, understanding curcumin's mechanism of action may elucidate compounds that would further improve it's inhibitory activity in an additive or synergistic manner leading to combination studies involving curcumin and other anti-viral agents and/or anti-fibrotic agents. A water soluble curcumin complex has recently been described that attenuated multiple markers of inflammation and injury, including pulmonary edema and neutrophil infiltration in an ALI model [86]. An additional study demonstrated the potential use of curcumin as an adjunct therapy as an anti-inflammatory, immunomodulatory agent along with antibiotics in the case of acute lung infection induced by K. pneumonia [87]. We are currently conducting additional experiments to characterize the mechanism of curcumin's affect on fibrotic lesion development in a therapeutic model. We have preliminary histological data that demonstrates treatment of reovirus 1/L-ALI/ARDS mice with curcumin beginning on day 7 post reovirus 1/L infection (after the onset of inflammation but before significant development of fibrotic lesions) inhibits fibrotic lesion development as evaluated on day 14 (unpublished results). Thus, continued studies of the potent anti-oxidant, anti-microbial, anti-inflammatory agent, curcumin, will likely improve the identification of key study endpoints that may be manipulated to slow or reverse the relentless progression of pulmonary inflammation/fibrosis in acute viral-induced ALI/ARDS, ultimately, leading to new novel treatments to reduce pulmonary dysfunction and improve outcome for critically ill patients.
