# Correction: Curcumin Modulates the Inflammatory Response and Inhibits Subsequent Fibrosis in a Mouse Model of Viral-induced Acute Respiratory Distress Syndrome

**DOI:** 10.1371/journal.pone.0134982

**Published:** 2015-08-04

**Authors:** Sreedevi Avasarala, Fangfang Zhang, Guangliang Liu, Ruixue Wang, Steven D. London, Lucille London

In Fig 4A, as the result of an error in preparation of the Western blot images, incorrect bands were shown for ß-actin, saline-treated α-SMA and saline-treated Tenascin. Bands labelled ß-actin were taken from the α-SMA blot by mistake; the band labelled saline-treated α-SMA was taken from a different lane showing curcumin-treated lysate in the same blot; the band labelled saline-treated Tenascin was from a different blot.

**Fig 4:**
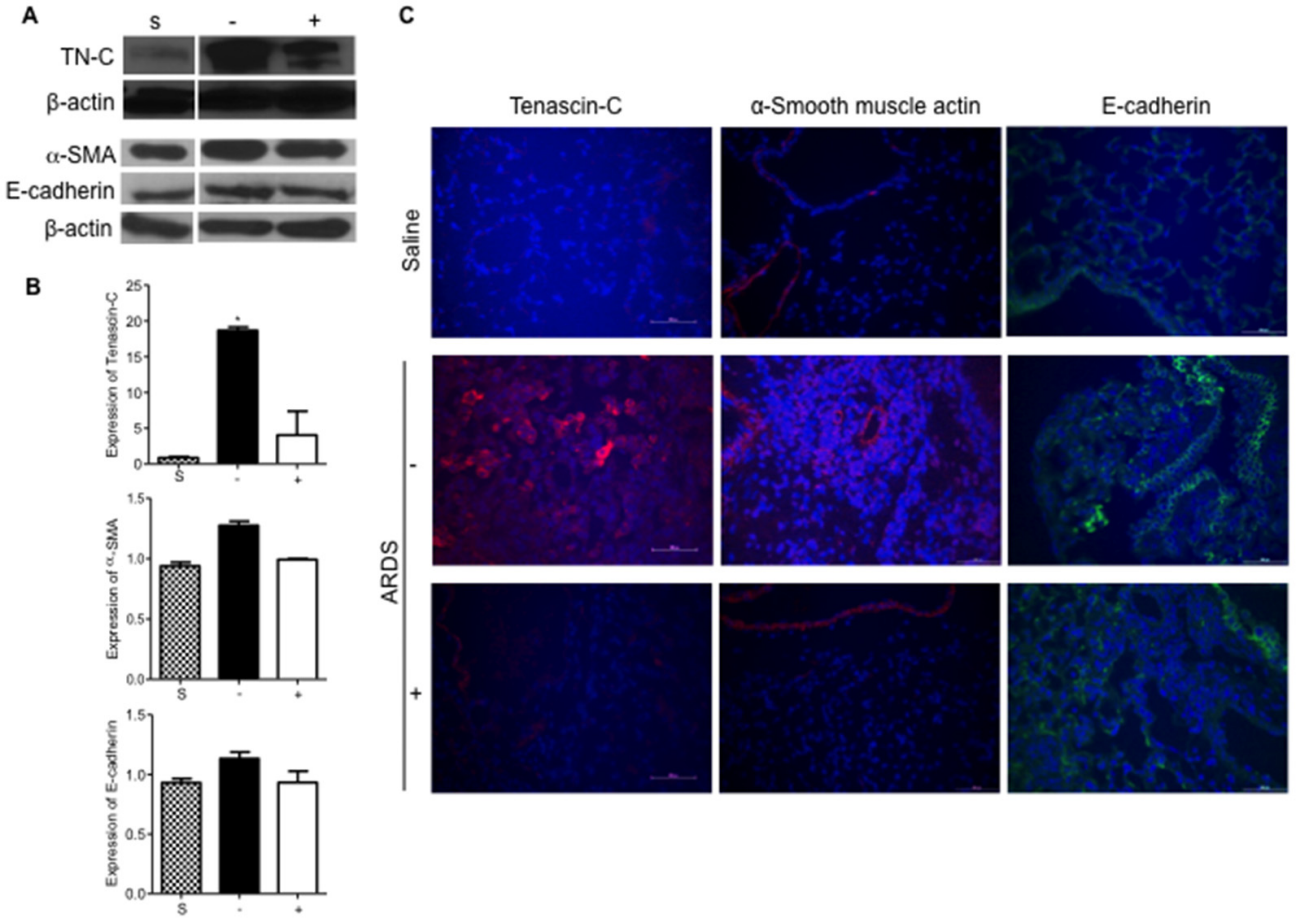
Administration of curcumin reduces expression of the myofibroblast cell phenotype in reovirus 1/L-ALI/ARDS. CBA/J mice were inoculated i.n. with 107 PFU reovirus 1/L and were either untreated (-) or treated (+) with 50 mg/kg curcumin by i.p. injection beginning 5 days prior to infection and daily, thereafter. (A) Western analysis from whole lung lysates for protein expression of TN-C, α-SMA, and E-cadherin from either saline (S), untreated (-), or curcumin-treated (+) reovirus 1/L-ALI/ARDS mice on day 14 post-inoculation. ß-actin expression demonstrated equal loading (Independent blots for TN-C and α-SMA/E-cadherin). Representative of three mice per time point; (B) RNA was prepared from whole lung tissue on day 14 post-inoculation and the relative expression of TN-C, α-SMA, and E-cadherin was assessed by qRT-PCR from untreated (-, solid bars) or treated (+, open bars) reovirus 1/L-ALI/ARDS mice. Saline inoculated mice were used as controls (S, stippled bars). Histograms are the mean +/- S.D. of three mice per time point. *p.

Additionally, α-SMA, E-cadherin and ß-actin expression were measured on a single blot that was stripped and re-probed multiple times, while Tenascin (TN-C) was probed on a different blot in an independent experiment with ß-actin antibodies used concurrently. The ß-actin bands from the independent Tenascin blot were not shown in the original Fig 4A.

Here we provide a revised Fig 4 showing the correct ß-actin and α-SMA bands, and a revised Fig 4 legend. The original uncropped blot images are provided as a supplementary file, with arrows indicating the correct sized bands. Multiple exposures are provided for the E-cadherin blot. The revised figure has been prepared using lanes 1, 6, and 7 of the original E-cadherin blot to match the lanes used from the ß-actin and α-SMA blots; the original published figure used lanes 1, 4, and 5 (lanes 4 and 6 both used lysates from untreated mice on day 14; lanes 5 and 7 both used lysates from curcumin-treated mice on day 14).

The errors in the original figure do not affect the results demonstrated in Fig 4A, which are further confirmed by the additional methods shown in Fig 4B and 4C.

## Supporting Information

S1 File Raw blots for Fig 4. (PPTX)
