# Supplementary material for: Correction: Curcumin Modulates the Inflammatory Response and Inhibits Subsequent Fibrosis in a Mouse Model of Viral-induced Acute Respiratory Distress Syndrome
Source: PLoS One. 2015 Aug 4;10(8):e0134982. doi: 10.1371/journal.pone.0134982 (PMC4524634; doi:10.1371/journal.pone.0134982)
Supplement: S1 File — (PPTX) [file pone.0134982.s001.pptx]

## Slide 1
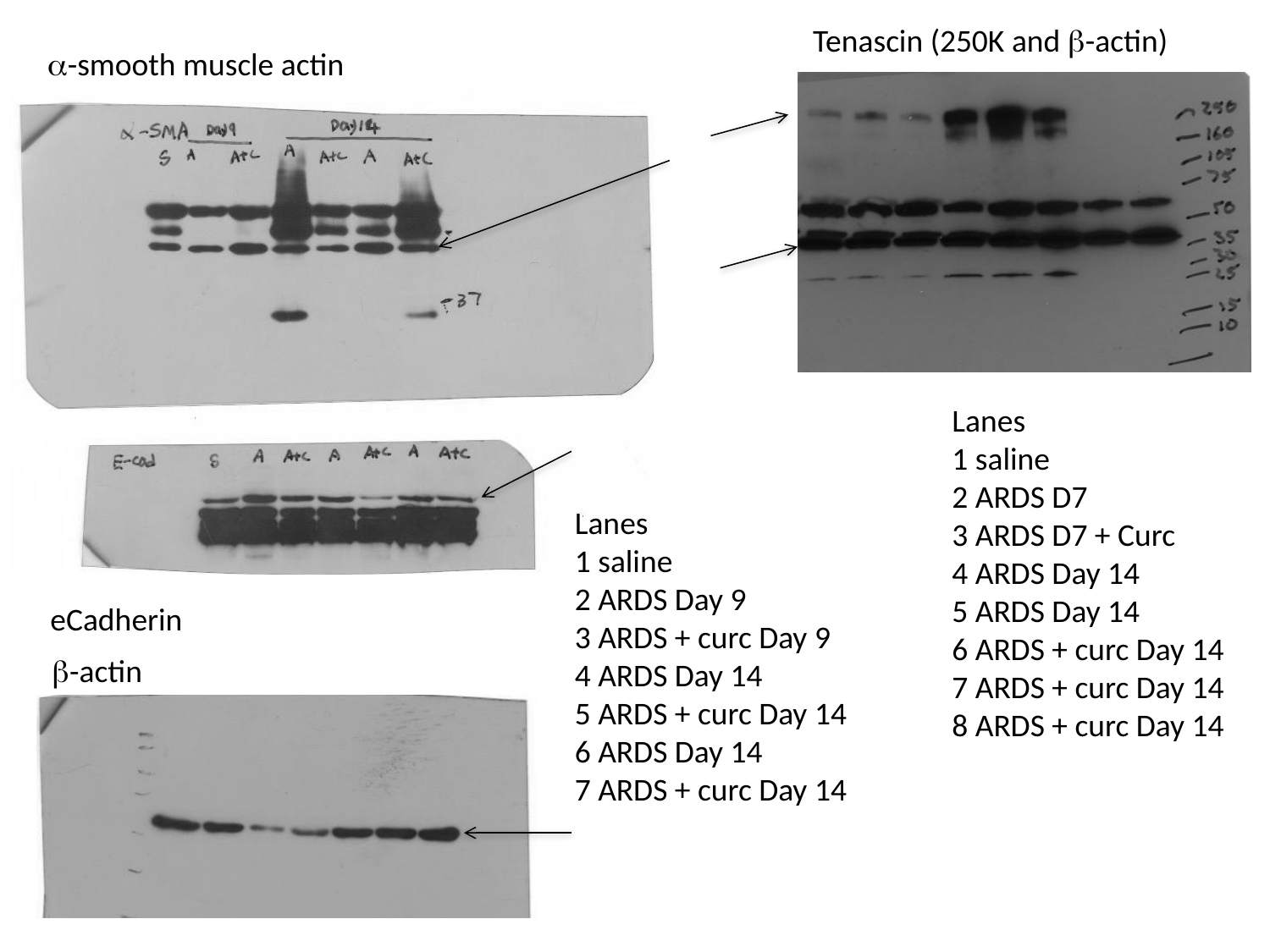

Tenascin (250K and b-actin)
a-smooth muscle actin
Lanes
1 saline
2 ARDS D7
3 ARDS D7 + Curc
4 ARDS Day 14
5 ARDS Day 14
6 ARDS + curc Day 14
7 ARDS + curc Day 14
8 ARDS + curc Day 14
Lanes
1 saline
2 ARDS Day 9
3 ARDS + curc Day 9
4 ARDS Day 14
5 ARDS + curc Day 14
6 ARDS Day 14
7 ARDS + curc Day 14
eCadherin
b-actin

## Slide 2
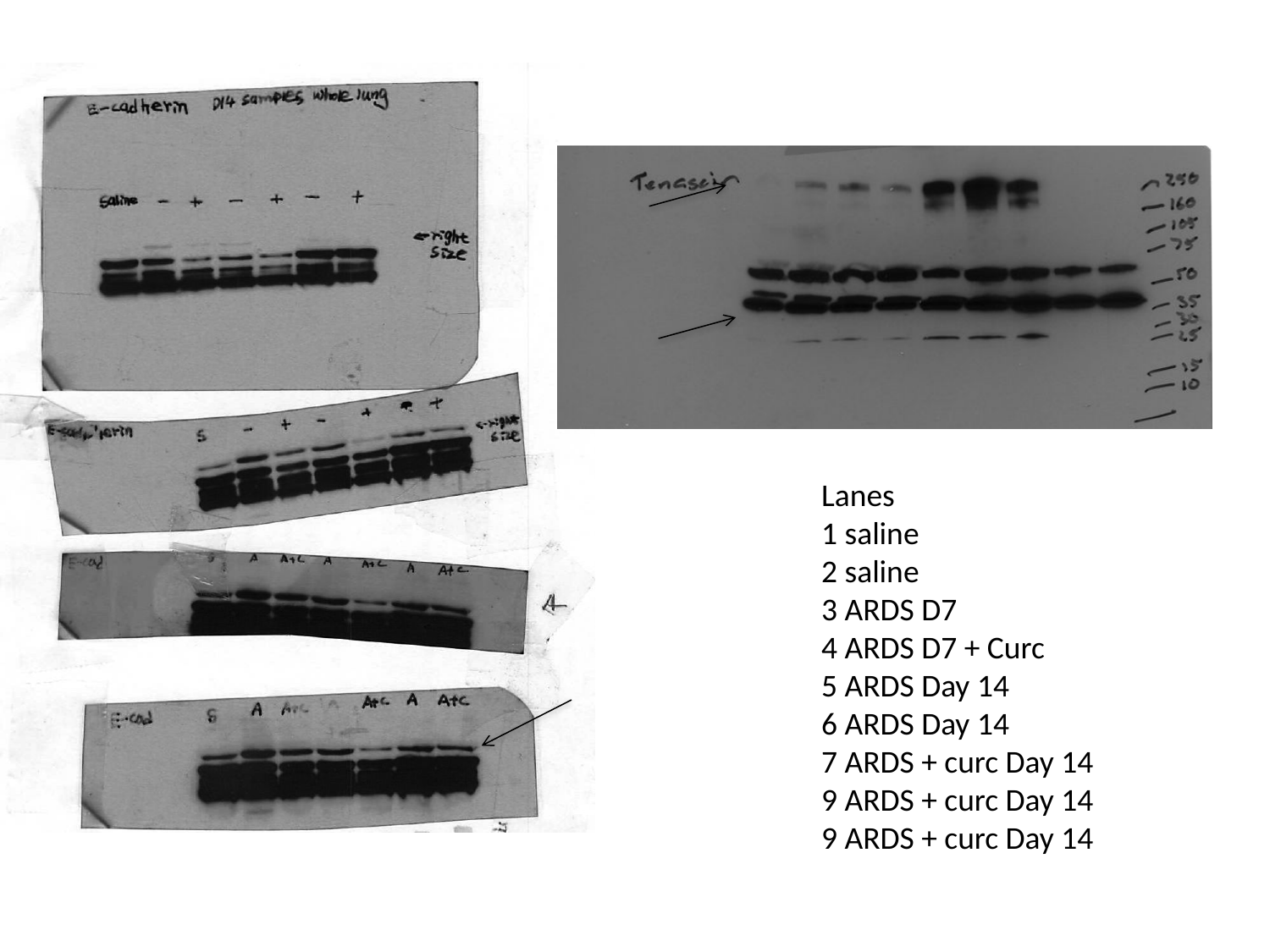

Lanes
1 saline
2 saline
3 ARDS D7
4 ARDS D7 + Curc
5 ARDS Day 14
6 ARDS Day 14
7 ARDS + curc Day 14
9 ARDS + curc Day 14
9 ARDS + curc Day 14
